# Analysis of clinicopathological characteristics and prognosis on primary gastric adenosquamous carcinoma

**DOI:** 10.1038/s41598-024-66701-x

**Published:** 2024-07-13

**Authors:** Yuqiang Du, Hongkun Tian, Zhiliang Chen, Gan Mao, Qian Shen, Qi Jiang, Yuping Yin, Kaixiong Tao, Xiangyu Zeng, Peng Zhang

**Affiliations:** Department of Gastrointestinal Surgery, Union Hospital, Tongji Medical College, Huazhong University of Science and Technology, No.1277 Jiefang Avenue, Wuhan, 430022 China

**Keywords:** Adenosquamous carcinoma, Component, Stomach, Clinicopathologic, Survival, Treatments, Gastric cancer, Prognostic markers

## Abstract

Primary gastric adenosquamous carcinoma (PGASC) is a rare type of gastric cancer with limited research and poorly understood clinicopathological features. This study investigated the clinicopathological features and outcomes of PGASC. Patients with PGASC from Union Hospital, Tongji Medical College, Huazhong University of Science and Technology and from the published literature were enrolled in this study. Survival curves were generated using the Kaplan–Meier method, and prognostic factors were identified through Cox proportional hazards regression models. This study identified 76 eligible cases of PGASC, with 45 cases from published literature and 31 from our center. The most prevalent symptoms were abdominal pain and dysphagia, with a median age of 62 years (range: 29–84 years). The primary lesions were predominantly in the proximal stomach, with a median tumor size of 6.5 cm (range: 1.5–16.0 cm). Tumor stages II, III, and IV were detected in 12 (16.7%), 43 (59.7%), and 17 (23.6%) patients, respectively. Most tumors were poorly differentiated in both the squamous cell carcinoma (SCC) component and adenocarcinoma (AC) component. The median survival time was 17 months (range: 2–122 months). The 1, 3, and 5-year overall survival (OS) was 60.7%, 31.1%, and 24.1%, respectively. Multivariate analysis revealed that OS was independently predicted by the proportion of SCC component, differentiation of AC component, and tumor stage. PGASC is a rare disease with a poor prognosis. A high proportion of SCC components, low differentiated AC components, and advanced tumor were associated with worse survival in patients with PGASC. Adjuvant therapy did not improve survival time.

## Introduction

Primary gastric adenosquamous carcinoma (PGASC) is an extremely rare subtype of gastric cancer characterized by combinations of two malignant components: adenocarcinoma (AC) and squamous cell carcinoma (SCC). It constitutes less than 1% of all gastric cancers worldwide and mostly affects the Asinas^1,2^. According to the Japanese Gastric Cancer Association, diagnosing PGASC requires the coexistence of AC and SCC in the primary tumor, with the latter comprising at least 25% of the tumor mass^3^. Gastric adenocarcinoma has long been a subject of intense research interest, with the development of effective systemic treatments being a key area of investigation. Currently, chemotherapy, radiotherapy, surgery, and immunotherapy have reached a relatively mature stage of development, with prognostic indicators such as albumin, the number of metastatic lymph nodes, and immune-related adverse events having been extensively explored and playing a pivotal role in guiding clinical treatment^4–6^. Compared to typical gastric adenocarcinoma, GASC appears to exhibit more aggressive clinicopathological features and poorer prognosis. The biological behavior of GASC may be determined by the AC component^7,8^. However, owing to the rarity of these carcinomas, PGASC has mostly been described in case reports and case series; therefore, various issues regarding PGASC remain unclear, including clinicopathological characteristics, optimal treatment strategies, and prognosis.

Therefore, in this retrospective study, we investigated the clinicopathological features, treatment, and survival outcomes of 76 patients with PGASC who underwent surgery. The aim was to summarize the specific biological behaviors and clinical features of PGASC, promoting a deeper understanding of this tumor and facilitating improved management.

## Patients and methods

### Study population

Cases of PGASC were collected from Union Hospital, Tongji Medical College, Huazhong University of Science and Technology, and published literature. Between 2011 and 2023, 5308 patients were diagnosed with gastric cancer in our hospital. Among them, 35 (0.7%) were diagnosed with GASC, and 31 were identified and analyzed. All patients underwent examination through contrast-enhanced computed tomography of the chest and abdomen, electronic or ultrasonic gastroscopy, tumor markers, renal and liver function, and other routine tests before surgery. All surgical samples were reviewed by at least one senior pathologist at our institution. We conducted a PubMed literature search for articles published between 1986 and 2023^8–18^. The search terms used were “adenosquamous AND stomach” OR “adenosquamous AND gastric” OR “carcinoma with squamous component AND stomach” OR “carcinoma with squamous component AND gastric.” Patients diagnosed with gastric AC with a squamous component, based on histology, were enrolled in this study. Inclusion criteria were as follows: age at diagnosis ≥ 18 years; confirmation of the diagnosis through the tumor tissue sample from surgery but not by autopsy or death certificate; and only one malignant primary tumor diagnosed. Patients with incomplete survival data or other necessary information were excluded.

This prospective study was approved by the institutional review board of Union Hospital, Tongji Medical College, Huazhong University of Science and Technology (Approval No. UHCT21823) and was conducted in accordance with the 1964 Helsinki Declaration and its later amendments or comparable ethical standards. The data were anonymous and, therefore, the requirement for informed consent was waived by the institutional review board of Union Hospital, Tongji Medical College, Huazhong University of Science and Technology.

### Data collection

Data on sex, age at diagnosis, clinical symptoms, tumor size, tumor site, differentiation grade, Borrmann classification, invasion depth, nodal status, tumor stage, the proportion of SCC components, lymphovascular invasion, perineural invasion, surgical complications, adjuvant therapy, and survival were collected. Gastric cancer staging was determined according to the eighth edition of the American Joint Committee on Cancer/Union for International Cancer Control tumor, node, and metastasis classification^19^. All surgical samples were reviewed by at least two senior pathologists from our institution. According to the Japanese Gastric Cancer Association (JGCA), a diagnosis of PGASC was confirmed when the characteristics of coexistence of AC and SCC components were identified, with the latter comprising at least 25% of the tumor mass^3^. Furthermore, immunohistochemistry staining showed that the tumor cells of the squamous carcinoma component expressed P63 and/or CK5/6, while the tumor cells of the adenocarcinoma component expressed CK7, CK20 and/or CK8/18. On gross pathology, downward invasion of the lower oesophagus to the cardia-gastric fundus was ruled out in all specimens.

The primary endpoint was overall survival (OS), defined as the duration from surgery to death from any cause, or to the last confirmed follow-up. Follow-ups were conducted via telephone or outpatient clinic visits. The cut-off date for follow-up was September 30, 2023.

### Statistical analysis

Univariate and multivariate Cox regression models were used to determine the prognostic relevance quantified as hazard ratios. Only factors that significantly influenced OS in the univariate Cox analysis were included in the multivariate Cox analysis. The OS rate was determined using the Kaplan–Meier method. The P value was considered statistically significant at 5% level. All statistical analyses were performed using the Statistical Package for the Social Sciences (IBM SPSS version 26.0; IBM Corp., Armonk, NY, USA) and GraphPad Prism Version 9.0.0 (GraphPad Software, San Diego, CA, USA; www.garphpad.com) for Windows.

### Ethics approval and consent to participate

Participants’ informed consent was not required for this study because of its retrospective nature. The Medical Ethics Committee of Union Hospital, Tongji Medical College, Huazhong University of Science and Technology approved this study, and the study was conducted in accordance with the Declaration of Helsinki.

## Results

### Patient characteristics

**Figure 1:**
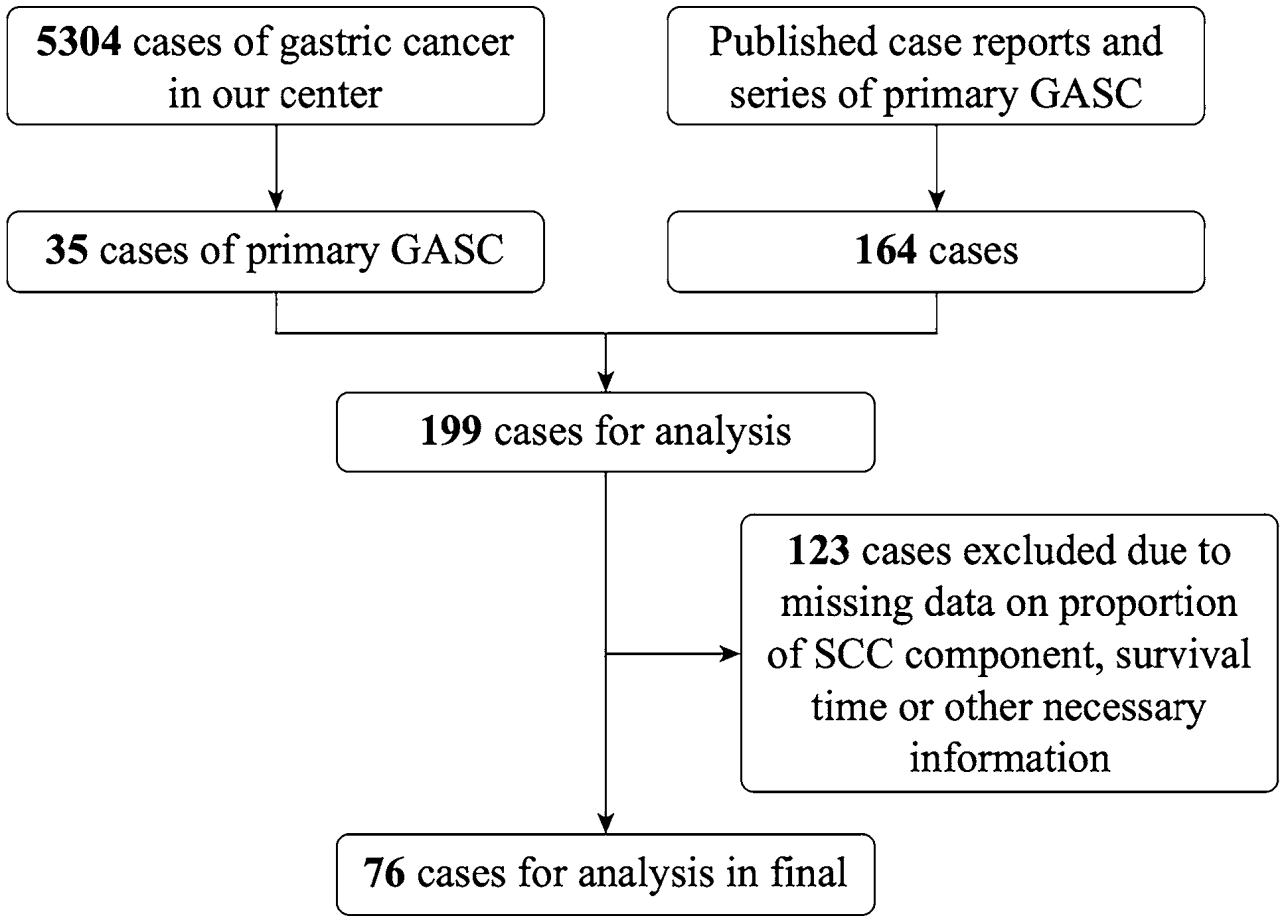
Flowchart of patient selection. GASC, gastric adenosquamous carcinoma; SCC, squamous cell carcinoma.

We identified 423 articles through a computerized search in PubMed as of September 2023. After applying the inclusion criteria, 23 articles were included in this study, providing information on 164 patients. Articles were collected through a manual review. With the addition of 35 additional patients from our center, 199 patients qualified for the study; however, only 76 were included. The exclusion of 123 cases was due to insufficient information on the proportion of the squamous component, survival time, or other necessary details (Fig. 1).

Previous literature reports and clinicopathological features of PGASC are summarized in Tables 1 and 2. Similar to gastric cancer, PGASC dose not present with specific clinical symptoms; abdominal pain and dysphagia are the primary symptoms in most patients. The median age of the 76 patients was 62 years (range: 29–84 years). Among them, 59 (77.6%) and 17 (22.4%) patients were male and female, respectively. Twelve patients had stage II disease, while those with stage III disease were 43. All patients had undergone radical gastrectomy for the first time. Patients with stage IV disease were 17, of whom 10 received neoadjuvant chemotherapy before surgery. Postoperative pathology showed that the primary site of the tumor was predominantly located in the proximal stomach, with a median tumor size of 6.5 cm (range: 1.5–16.0 cm). The SCC component proportion was divided into three categories: 25–50%, 50–75%, and > 75%. These categories were observed in 22 (28.9%), 23 (30.3%), and 31 (40.8%) patients, respectively. Most patients had lymph node metastases, and most tumors were poorly differentiated in both the SCC and AC components.

**Table 1:** The summary of primary gastric adenosquamous carcinoma from previous literature.

Author	Case	Age	Proportion of SCC (%)	Tumor stage	Survival months
Faria et al. ^6^	1	84	80	III	27
Fukai et al. ^14^	1	81	65	III	6
Alsheikh et al. ^13^	1	57	90	IV	4
Sun et al. ^11^	1	50	90	III	12
Bansal et al. ^4^	1	37	40	IV	2
Ajoodhea et al.	1	70	60	III	20
Saito et al. ^15^	4	58–80	25–80	II–IV	6–118
Chen et al. ^9^	7	47–75	40–90	III–IV	2–47
Chen et al. ^1^	12	43–79	25–75	II–IV	5–52
Mori et al. ^10^	16	32–78	25–75	II–IV	2–64

SCC, squamous cell carcinoma.

**Table 2:** Clinicopathological features of primary gastric adenosquamous carcinoma.

Character	n (%)	Character	n (%)
Sex		Borrmann classification	
Male	59 (77.6)	I/II	36 (47.4)
Female	17 (22.4)	III/IV	26 (34.2)
Age (year)		Lymph node metastasis	
≤ 60	31 (40.8)	Negative	13 (17.1)
> 60	45 (59.2)	Positive	63 (82.9)
Tumor site		Perineural invasion	
U	43 (56.6)	Negative	16 (21.1)
M	12 (15.8)	Positive	35 (46.1)
L	21 (28.9)	LVI	
Tumor size (cm)		Negative	22 (28.9)
≤ 5	24 (31.6)	Positive	30 (39.5)
> 5	51 (67.1)	Tumor stage	
Proportion of SCC component, %		II	12 (16.7)
25–50	22 (28.9)	III	43 (59.7)
50–75	23 (30.3)	IV	17 (23.6)
> 75	31 (40.8)	Adjuvant therapy	
Differentiation of SCC component		No	21 (27.6)
Low	41 (53.9)	Yes	39 (51.3)
Moderately/Well	18 (23.7)	Complication	
Differentiation of AC component		No	22 (28.9)
Low	41 (53.9)	Yes	28 (36.9)
Moderately/Well	18 (23.7)		
Tumor invasion			
T2–3	36 (47.4)		
T4a	19 (25.0)		
T4b	21 (27.6)		

AC, adenocarcinoma; L, lower third of the stomach; LVI, lymphovascular invasion; M, middle third of the stomach; SCC, squamous cell carcinoma; U, upper third of the stomach.

### Survival outcome

**Figure 2:**
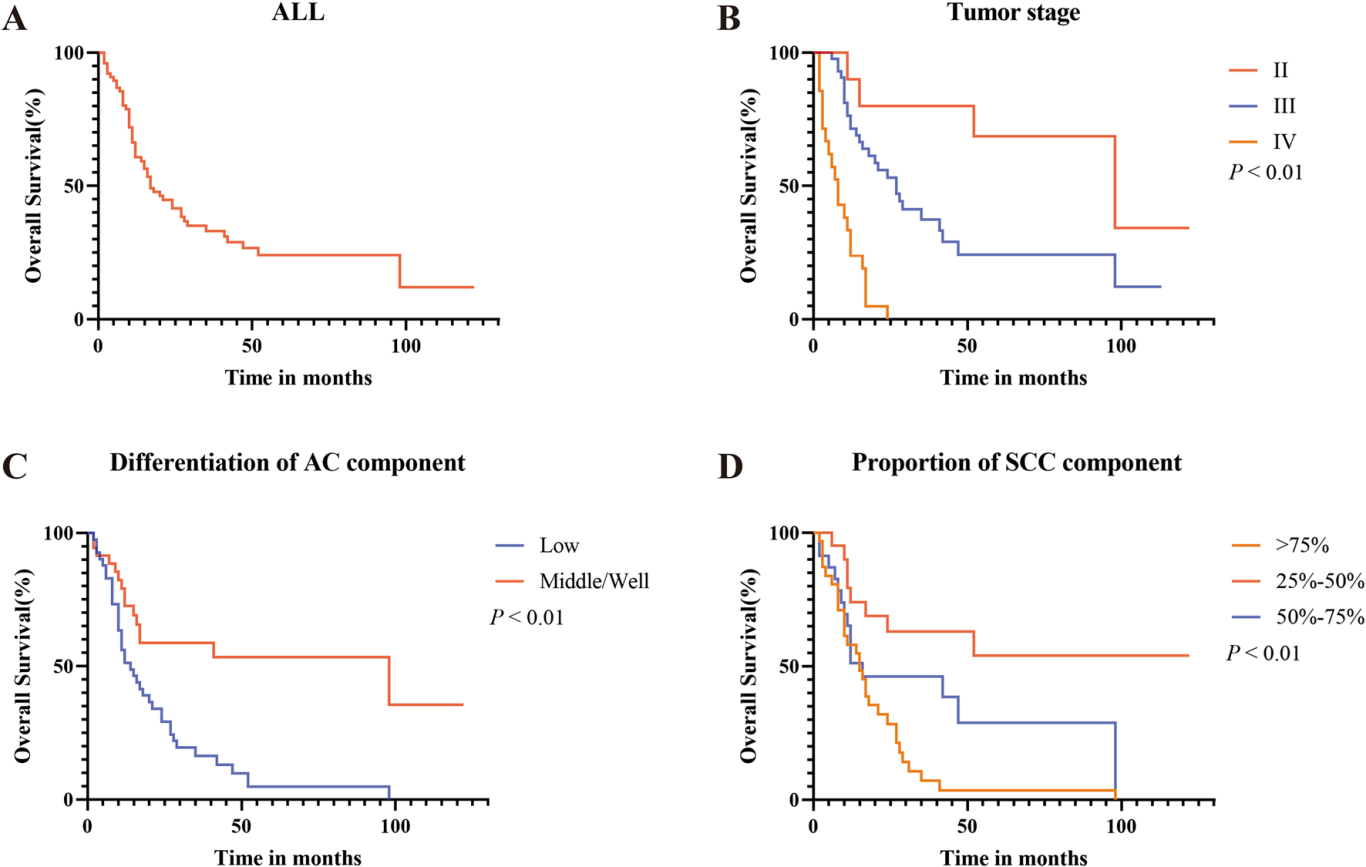
Survival analysis of gastric adenosquamous carcinoma. (**A**) Overall survival curves for all patients with primary gastric adenosquamous carcinoma. (**B**–**D**) Overall survival curves for patients with primary gastric adenosquamous carcinoma based on tumor stage (**B**), differentiation of adenocarcinoma cell component (**C**) or proportion of squamous cell component (**D**).

All 76 patients were included in the survival analysis. The median survival time was 17 months (range: 2–122 months). The 1-, 3-, and 5-year OS rates were 60.7%, 31.1%, and 24.1%, respectively (Fig. 2). Table 3 shows the results of the univariate and multivariate Cox regression analyses. The univariate analyses revealed that OS was significantly associated with a proportion of the SCC component (*p* = 0.001), differentiation of SCC component (*p* = 0.001), differentiation of AC component (*p* = 0.001), invasion depth (*p* = 0.032), tumor stage (*p* = 0.001), lymph node metastasis (*p* = 0.001), Borrmann classification (*p* = 0.03), perineural invasion (*p* = 0.002), LVI (*p* = 0.02), and adjuvant therapy (*p* = 0.005). Furthermore, the multivariate analysis revealed that OS was independently predicted by a proportion of the SCC component (HR: 98.575; 95% CI: 5.408–1796.77; *p* = 0.002), differentiation of AC component (HR: 0.028; 95% CI: 0.002–0.351; *p* = 0.006), and tumor stage (HR: 608.83; 95% CI: 11.96–30,983.7; *p* = 0.001). The OS stratified by the proportion of SCC components, differentiation of AC components, and tumor stage are shown in Fig. 2.

**Table 3:** Univariate and multivariate analysis of overall survival for the primary gastric adenosquamous carcinoma.

Clinicopathological factors	Univariate analysis			Multivariable analysis		
	HR	95% CI	*P* value	HR	95% CI	*P* value
Sex (Male/Female)	0.694	0.349–1.383	0.299			
Age (> 60/ ≤ 60)	1.004	0.580–1.737	0.989			
Tumor site (Proximal/Distal/Most + Total)	1.125	0.584–2.166	0.725			
Proportion of SCC component (25–50%/50–75%/ > 75%)	4.309	1.936–9.556	0.001	98.57	5.408–1796	0.002
Tumor size (> 5.0 cm/ ≤ 5.0 cm)	1.694	0.914–3.137	0.094			
Differentiation of SCC component (Low/Moderately + Well)	0.186	0.072–0.484	0.001	0.33	0.041–2.649	0.297
Differentiation of AC component (Low/Moderately + Well)	0.055	0.013–0.234	0.001	0.028	0.002–0.351	0.006
Tumor stage (II/ III/ IV)	20.01	6.010–66.64	0.001	60.88	11.96–309.8	0.001
Invasion depth (T2 + T3/T4a/T4b)	2.149	1.070–4.317	0.032	3.053	0.422–22.07	0.269
Borrmann classification (I + II/III + IV)	0.488	0.255–0.931	0.03	0.695	0.059–8.194	0.772
Lymph node metastasis (Positive/Negative)	3.902	1.384–11.00	0.01	1.916	0.110–33.38	0.656
Perineural invasion (Positive/Negative)	3.159	1.509–6.615	0.002	1.486	0.145–15.23	0.739
LVI (Positive/Negative)	2.712	1.167–6.305	0.020	14.88	0.507–437.0	0.117
Adjuvant therapy (No/Yes)	0.397	0.208–0.757	0.005	0.394	0.077–2.030	0.266
Complication (No/Yes)	1.781	0.879–3.611	0.109			

HR, Hazard ratio; SCC, squamous cell carcinoma; AC, adenocarcinoma; LVI, lymphovascular invasion.

## Discussion

The incidence of PGASC is extremely low, comprising less than 1% of all gastric malignancies and approximately 2.2% of all adenosquamous carcinomas^20^. The clinicopathological features and prognosis of PGASC have been investigated in several previous studies. In the present study, the median age of patients with PGASC was 62 years, similar to that reported by Feng et al. with a mean age of 61.3 years^1^. The prevalence of PGASC was higher in older populations, with a male/female ratio of 3.47, as per previous studies, indicating that more males than females may be affected. While some studies have indicated that the lower third of the stomach is the most common site for PGASC^1,21,22^, our findings suggest that the majority of tumors were located in the proximal stomach, consistent with some recent registry database studies^2,23–25^. The disparate outcomes may be attributable to a multitude of variables, including ethnicity, dietary patterns, and the living environment.

The clinical manifestations of PGASC are similar to those of primary gastric cancer, including abdominal pain, dysphagia, and nausea^2^. However, PGASC is more prone to exhibiting known aggressive behaviors, including poor differentiation, advanced staging at diagnosis, lymphovascular and perineural invasion, and lymph node metastases, potentially explaining its poor prognosis^1,2,22,25,26^. In this study, the median survival time was 17 months, slightly shorter than that reported in previous studies, which ranged from 12 to 24 months^2,27^. Although numerous studies have been conducted, the prognostic factors of PGASC remain obscure. In our study, we found that the proportion of the SCC component, differentiation of the AC component, and tumor stage were independent risk factors for prognosis.

However, the role of the SCC component in the survival of patients with PGASC remains unclear. While, SCC is theoretically associated with a less favorable survival rate than conventional gastric AC^12,28^, some researchers have suggested that biological behaviors may be determined by AC rather than SCC in PGASC. The AC component is predominantly found in hematogenous and hepatic metastases, and the biological behavior of GASC is usually similar to that of an aggressive adenocarcinoma^1,2,29^. However, there is limited research on the correlation between various AC/SCC components and survival rates in patients with PGASC. Li et al.^21^ performed a clinicopathological analysis of 45 cases and reported that with an increase in the proportion of the SCC component (range, 15–85%), the 5-year survival rate gradually decreased (range, 17.2–0%), which was not the observed with the AC component. Moreover, the hazard plot showed that the risk of death gradually increased with an increase in the proportion of the SCC component in the primary lesion. Based on a real-world setting and relatively large sample size, our findings indicate that patients with a high proportion of the SCC component (> 75%) have a significantly shorter OS than those with a small or medium proportion, proving that the SCC component is more malignant than the AC component.

Tumor staging is an important factor in disease prognosis. In the current study, multivariate analysis suggested that tumor stage was independently related to prognosis. These findings were consistent with those of previous studies^1,23,30^. Kaplan–Meier analysis revealed that patients with deeper tumor invasion had shorter OS. Nonetheless, no significant difference was found in multivariate Cox analysis, likely attributable to the small sample size and low statistical power.

In terms of tumor differentiation, patients with undifferentiated or poorly differentiated gastric cancers present a greater risk than those with moderately or well-differentiated cancers^26^. We observed differences in the pathological differentiation of the AC and SCC components in patients with PGASCs. Furthermore, our results revealed that the pathological differentiation of the AC component significantly influenced prognosis, whereas that of the SCC component did not. To our knowledge, this is the first study to evaluate the pathological differentiation grade of different components in adenosquamous carcinoma, suggesting the need to consider not only the proportion of different components but also the pathological differentiation grade of different components in future treatment.

Currently, no standard treatment strategy has been established for PGASC. Radical resection remains the optimal treatment for local disease without distant metastasis, and the benefits of chemotherapy and radiotherapy as adjuvant treatments remain unclear^13,23^. In our study, we observed a trend toward better survival in patients treated with adjuvant therapy (median survival time, 42–15 months). However, adjuvant therapy was not a prognostic factor in the multivariate analysis. Our study demonstrated that both the AC component and the SCC component in PGASC affect the prognosis. It was hypothesised that the adjuvant radiotherapy in combination with chemotherapy would be more effective than radiotherapy alone or chemotherapy alone. However, due to the small number of cases in this paper and the lack of specific differentiation between adjuvant treatment scenarios, it was not possible to draw the appropriate conclusions. Furthermore, recent studies have indicated that patients with PGASC are more likely to exhibit positive results for CPS and dMMR, suggesting that immunotherapy can be considered as frontline systemic treatment^31^. In the future, it will be beneficial to explore the potential of multiple treatment modalities to improve the prognosis of patients with adenosquamous carcinoma.

This study has a few limitations. First, the sample size was not large enough due to the rarity of the disease; therefore, the results of our study should be interpreted with caution. However, this study represents the largest number of PGASC cases with detailed pathological information to date. Second, the retrospective nature of the study cannot exclude the possibility of a selection bias, although we have rigorously adhered to uniform criteria for case inclusion. Third, there were limited data on gene mutations, targeted therapy, and cancer recurrence, which may have influenced the findings of the survival analysis. Despite these limitations, our results may contribute to future studies on PGASC. Our study summarized the clinicopathological features and prognostic survival of PGASC and identify a number of relatively reliable factors associated with long-term prognosis. Clinicians can benefit from these predictors in identifying high-risk patients and subsequently facilitating follow-up studies. Further molecular mechanism studies can be conducted to investigate the proportion of SCC and the differentiation of AC component in adenosquamous carcinoma. This will enable the formulation of individualised treatment plans for patients with different proportions of different differentiation, with a view to improving the survival time of this group of patients. Ongoing research will continue to enhance our understanding of this rare disease.

## Conclusion

Our study showed that PGASC is a rare disease with a poor prognosis. Patients with a high proportion of SCC, a poorly differentiated AC component, and an advanced tumor stage have worse survival rates. However, adjuvant therapy did not improve patient survival. These findings can guide clinicians in developing tailored treatment approaches and improve the overall understanding of this rare gastric malignancy. Large-sample prospective controlled clinical studies are needed to validate these conclusions.

## Data Availability

The datasets used in this study are available from the corresponding author on reasonable request.

## Abbreviations

GASC: Gastric adenosquamous carcinoma
AC: Adenocarcinoma
SCC: Squamous cell carcinoma
LVI: Lymphovascular invasion
OS: Overall survival
HR: Hazard ratio
